# A Novel 3D Hierarchical Plasmonic Functional Cu@Co_3_O_4_@Ag Array as Intelligent SERS Sensing Platform with Trace Droplet Rapid Detection Ability for Pesticide Residue Detection on Fruits and Vegetables

**DOI:** 10.3390/nano11123460

**Published:** 2021-12-20

**Authors:** Guanliang Sun, Ning Li, Dan Wang, Guanchen Xu, Xingshuang Zhang, Hongyu Gong, Dongwei Li, Yong Li, Huaipeng Pang, Meng Gao, Xiu Liang

**Affiliations:** 1 Key Laboratory for High Strength Lightweight Metallic Materials of Shandong Province (HM), Advanced Materials Institute, Qilu University of Technology (Shandong Academy of Sciences), Jinan 250014, China; 13793994223@163.com (G.S.); lining3875@163.com (N.L.); wangdan1910@163.com (D.W.); gcxu@sdas.org (G.X.); xszhang@qlu.edu.cn (X.Z.); hygong@sdas.org (H.G.); dwli@sdas.org (D.L.); yongli@sdas.org (Y.L.); p5415cl@163.com (H.P.); mgao@sdas.org (M.G.); 2 Center of Excellence for Environmental Safety and Biological Effects, Beijing Key Laboratory for Green Catalysis and Separation, Department of Chemistry and Biology, Beijing University of Technology, Beijing 100124, China

**Keywords:** surface-enhanced Raman scattering, Cu@Co_3_O_4_@Ag, trace droplet, pesticide residues, 3D hierarchical plasmonic nanomaterials, Cu foam framework

## Abstract

Rapid and effective detection of pesticide residues from complex surfaces of fruits and vegetables has important significance. Herein, we report a novel three-dimensional (3D) hierarchical porous functional surface-enhanced Raman scattering (SERS) substrate, which is fabricated by successive two-step hydrothermal synthesis strategy of silver nanoparticles (Ag NPs) and cobalt oxide nanowires (Co_3_O_4_ NWs) on the 3D copper foam framework as Cu@Co_3_O_4_@Ag-H. The strategy offers a new avenue for localized plasmonic materials distribution and construction, which exhibits better morphology regulation ability and SERS activity (or hotspots engineering) than physical spurring obtained Cu@Co_3_O_4_@Ag-S. The developed Cu@Co_3_O_4_@Ag-H possesses large surface area and rich hotspots, which contributes to the excellent SERS performance, including homogeneity (RSD of 7.8%), sensitivity (enhancement factor, EF of 2.24 × 10^8^) and stability. The Cu@Co_3_O_4_@Ag-H not only provides plenty of Electromagnetic enhancement (EM) hotspots but also the trace detection capability for droplet rapid sensing within 2 s. Cu@Co_3_O_4_@Ag-H substrate is further developed as an effective SERS sensing platform for pesticide residues detection on the surfaces of fruits and vegetables with excellent LOD of 0.1 ppm, which is lower than the most similar reported works. This work offers new potential for bioassay, disease POCT diagnosis, national security, wearable flexible devices, energy storage and other related fields.

## 1. Introduction

Pesticide residues have become a worldwide problem in the field of food safety. It is an urgent problem to develop fast and effective pesticide residue detection methods to assist in the supervision of food safety [1]. So far, there have been many detection methods for pesticide residues, such as enzyme-linked immunosorbent assay (ELISA), enzyme inhibition and Liquid chromatography-mass spectrometry (LC-MS) [2,3,4]. However, these detection methods are expensive, time-consuming and complex, and cannot give consideration to both rapidity and sensitivity. Surface enhanced Raman scattering (SERS), a spectral detection technique, has been widely used in environmental monitoring [5], biomedicine [6,7], food safety [8], public safety [9] and other fields [10] in recent years for its excellent sensitivity, ultra-fast detection speed and nondestructive detection. SERS technique is capable of specific “diagnosis” based on the vibration patterns of different molecules. Compared with other detection methods, SERS technique can detect target molecules more quickly, accurately and sensitively [11]. Such superior performance of SERS is supported by the excellent SERS substrate. The substrate relies primarily on two enhancement modes: Electromagnetic Enhancement (EM) and Chemical Enhancement (CM) [12]. Since SERS was first observed on rough Ag electrodes [13,14,15], many efforts have been made to improve the enhancement effect. From a single noble metal particle with adjustable morphology [16,17] to a composite noble metal structure with adjustable shape, size and arrangement [18,19], and then to the development of noble metal semiconductor composite structure substrate [20,21], the improvement and breakthrough of higher sensitivity and detection efficiency are realized step by step, which also reflects the diversified development of SERS substrate.

SERS substrates of sols or two-dimensional (2D) plane structure, such as nanospheres, nanostars, nanorods sols, or tape modified with gold NPs are widely used [22,23,24,25,26]. 2D SERS substrate is simply loaded with a layer of nanoparticles, in which SERS hotspots exist most in the 2D plane. However, the laser confocal volume in the Raman device is a three-dimensional (3D) space [27,28,29]. The hotspots existing in the 2D plane can only enhance the limited pesticide molecular signal in the plane, and cannot make full use of the 3D laser confocal volume [30]. This results in fewer pesticide molecules forming the limited signal intensity and lower SERS sensitivity. In order to effectively use the 3D space of the confocal laser and enhance the Raman detection performance, researchers have explored a variety of SERS substrates with 3D structures [31,32,33]. In seeking a low-cost, easy-to-prepare 3D SERS substrate, we have explored the novel 3D SERS substrate based on copper foam. Copper foam is a new multifunctional material with a large number of connected or disconnected holes evenly distributed in its interior [34]. It has a high specific surface area and regular 3D structure, good ductility. It is mostly used as battery negative (carrier) material and catalyst carrier because of its abundant micron-scale pores [35]. It is prepared by electrodeposition and sintering method, which is low cost and can be prepared in bulk. Substrates of the desired size and shape can be obtained by simple cutting without changing the properties of the substrate. However, copper foam has low specific surface area and smooth surface, which is not conducive to capturing the target, and the SERS enhancement effect of copper is relatively low [36], so in order to obtain a better detection effect, we will modify the surface of copper foam with cobalt oxide nanowires (Co_3_O_4_ NWs) to construct a 3D multi-level structure. The large number of microscale gaps between the copper foam skeleton allows ample space for a variety of surface modifications, which offers the opportunity to design multi-stage hot spot systems to make full use of the 3D laser confocal volume. Moreover, the combination of Cu foam@Co_3_O_4_ NWs and precious metal particles can realize the synergistic enhancement effect of the substrate and greatly increase the specific surface area and detection sensitivity of the substrate.

Herein, in order to effectively utilize the laser confocal volume, we have explored a new plasmonic functional nanomaterial with 3D hotspot distribution as SERS substrate. A novel successive two-step hydrothermal synthesis strategy with good morphology regulation ability and SERS activity was developed to fabricate a novel 3D hierarchical porous Cu@Co_3_O_4_@Ag-H substrate. As the matrix layer of the substrate, foamed copper has a large specific surface area and flexibility. Co_3_O_4_ NWs grow densely on the surface of the foamed copper, providing copper foam a better chemical enhancement effect and a wider space for the growth of nanoparticles and the adsorption of pesticide molecules. Ag NPs are aligned evenly and tightly on the Cu@Co_3_O_4_ NWs to form 3D hotspots, which maximize the detection performance of the substrate and effectively adsorb pesticide molecules to the hotspots area. The developed Cu@Co_3_O_4_@Ag-H possesses large surface area and rich hotspots, which contributes to the excellent SERS performance, including homogeneity sensitivity and stability. Good morphology regulation ability and SERS activity were evidenced more than physical spurring to obtain Cu@Co_3_O_4_@Ag-S. Besides, the trace detection capability for droplet rapid sensing was achieved by superhydrophobic flexible Cu@Co_3_O_4_@Ag-F SERS sensor. More importantly, Cu@Co_3_O_4_@Ag-H substrate is further developed as effective SERS sensing platform for pesticide residues detection on fruits and vegetables surfaces with excellent sensitivity, which is far lower than the permitted dose in food safety. The Cu@Co_3_O_4_@Ag SERS substrate shows an excellent detection performance for trace chemicals and provides a new approach for future template-free 3D hierarchical porous functional materials synthesis as potential choices for bioassay, disease POCT diagnosis, national security, wearable flexible devices and related fields.

## 2. Materials and Methods

### 2.1. Materials

Silver nitrate (AgNO_3_, 99.99%) and ammonia (NH_3_·H_2_O), cobalt nitrate (Co(NO_3_)_2_·6H_2_O, 99%), urea (CH_4_N_2_O, 99.5%) and ammonium fluoride (NH_4_F, 99.99%) were purchased from the Aladdin Company (Shanghai, China). Super clean 3D foam copper was purchased from Kunshan Fei Meite electronic new material Co., Ltd (Suzhou, China). Thiram (C_6_H_12_N_2_S_4_, 97%), rhodamine 6G (R6G, C_28_H_31_N_2_O_3_Cl, 95%) and 4-nitrophenylthiophenol (4-NBT) were purchased from the Macklin Co., Ltd. (Shanghai, China). 1H, 1H, 2H, 2H perfluorooctyl trichlorosilane (FAS-17, 97%) was purchased from sigma Aldrich (Shanghai, China), Milli-Q water (>18.2 MΩ·cm at 25 °C) was employed in all experiments.

### 2.2. Characterizations

The synthesized Cu foam@Co_3_O_4_ NWs and Cu@Co_3_O_4_@Ag SERS substrate were characterized by SEM (JEOL, Japan, 15 kV), XRD (X’ Pert PRO MPD, The Netherlands, 5°/min, 10°–90°), Raman spectra were recorded from a Horiba JY iHR550 (France) system with excitation wavelength at 532 nm (~1.5 mW). During measurements, the laser beam was focused on a spot of 2 μm diameter by a microscope objective with a magnification of 50×. The contact angle is measured by the SINDIN contact angle measuring instrument (SDC-100, Qingdao, China), TEM and HRTEM images were acquired using a JEM-2100 electron microscopy (JEOL), with an Operating Voltage at 200 kV.

### 2.3. Synthesis of Cu Foam@Co_3_O_4_ NWs

The copper foam was cut into 3 × 3 cm^2^ squares, and then the 3D copper foam was pressed into thin slices of 0.02 mm thickness by pressing, ultrasonically cleaned for 5 min, and dried at 60 °C for 2 h.

Co_3_O_4_ NWs were synthesized by hydrothermal method according to the previous reports [37]. In a typical synthesis, 1.45 g of Co(NO_3_)_2_·6H_2_O was dissolved in 35 mL of deionized water and stirred at room temperature for 5 min. Next, the mixture of 0.92 g of NH_4_F and 1.51 g of CH_4_N_2_O was slowly added to the above solution and deionized water was added until the solution reached 48 mL. The solution was stirred at room temperature for 30 min. Then the reaction mixture was hydrothermally treated at 120 °C for 6 h. Upon completion of the reaction, squares were washed three times with deionized water and dried under vacuum for 12 h at 50 °C. Finally, the square was annealed for 180 min under nitrogen atmosphere at a heating rate of 3 °C min^−1^ to 430 °C, and the black Cu foam@Co_3_O_4_ NWs sample was obtained.

### 2.4. Synthesis of Hierarchical Cu@Co_3_O_4_@Ag Substrate

Cu@Co_3_O_4_@Ag were prepared following a previously reported protocol with a modified procedure [38]. Ammonia (1 wt%) was added dropwise to AgNO_3_ solution (50 mM) while stirring until the solution became turbid and then clarified and kept stirring for 30 min to obtain a silver-ammonia solution (40 mM). Next, Cu foam@Co_3_O_4_ NWs were immersed into the silver-ammonia solution and hydrothermally treated at 170 °C for 4 h. The flexible Cu@Co_3_O_4_@Ag-H substrates were washed and dried under vacuum at 40 °C for 12 h for further use.

The density and particle size of the Ag NPs within the structure Cu foam@Co_3_O_4_ NWs could be regulated by altering the concentration of silver ammonia solution (30 mM, 40 mM, 50 mM). The other reaction conditions were not changed, unless otherwise stated.

The Cu@Co_3_O_4_@Ag-S substrates were prepared by depositing different thicknesses of Ag on clean Cu foam@Co_3_O_4_ NWs and altering sputtering time (5 min, 10 min and 15 min) with diffusion mode in Leica EM ACE200 system.

### 2.5. Synthesis of Superhydrophobic Cu@Co_3_O_4_@Ag-F Substrates

The synthesis of superhydrophobic Cu@Co_3_O_4_@Ag-F substrate is carried out by surface hydrophobic treatment on the basis of the Cu@Co_3_O_4_@Ag-H substrate. The typical synthesis is as follows.

1 g of FAS-17 was added into 99 g of ethanol and stirred at 150 rpm for 3 h. The prepared Cu@Co_3_O_4_@Ag-H was immersed in the above solution at room temperature and left to stand for 12 h. The Cu@Co_3_O_4_@Ag-F was then washed several times with ethanol and dried under vacuum at 100 °C for 1 h for further use.

### 2.6. SERS and Raman Measurements

In the experiment, 10 μL (unless droplet detection of 1μL) of different concentrations of 4-NBT, R6G and thiram solutions were used to detect, and the Raman spectra were obtained using 532 nm laser for 4-NBT and R6G and 633 nm laser for thiram with 1.5 mW power for 5 s.

When testing the surface of fruits and vegetables for pesticide residues, four typical fruits and vegetables with different surface roughness of orange, cabbage, tomato and apple were selected as models for evaluation. Ten microliters of 100 ppb of thiram standard solution was added to a 1 cm^2^ area on their epidermis, then dried under ambient conditions. The surfaces were infiltrated with ethanol, wiped with substrate and detected.

## 3. Results and Discussion

### 3.1. Preparation and Characterization of Cu@Co_3_O_4_@Ag-H

**Figure 1:**
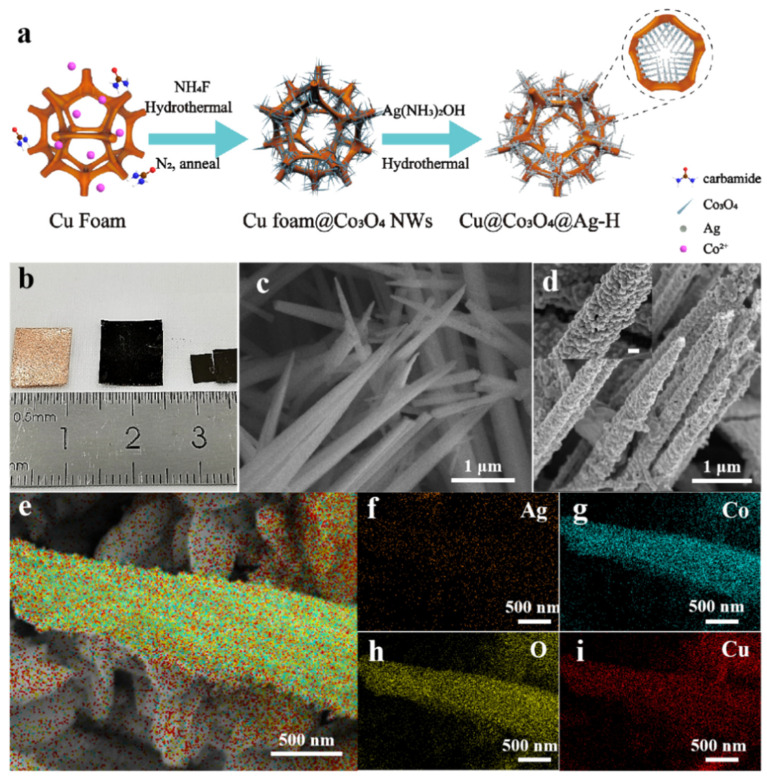
(**a**) Schematic illustration of the fabrication process toward hierarchical Cu@Co_3_O_4_@Ag-H substrate. (**b**) Photographs of Cu Foam (left), Cu@Co_3_O_4_ NWs (mid), Cu@Co_3_O_4_@Ag-H (right). (**c**,**d**) Corresponding SEM images of Cu@Co_3_O_4_ NWs and Cu@Co_3_O_4_@Ag-H. The scale bar in insert of (**d**) is 100 nm. (**e**–**i**) SEM elemental mappings of Cu@Co_3_O_4_@Ag-H: (**e**) the overlay selected square area EDS mapping distribution image and (**f**–**i**) corresponding mapping distribution images of Ag, Co, O and Cu elements.

Figure 1a presents a novel successive two-step hydrothermal synthesis strategy for flexible hierarchical Cu@Co_3_O_4_@Ag-H substrate. Figure 1b shows the optical pictures of the copper foam, Cu@Co_3_O_4_ NWs and Cu@Co_3_O_4_@Ag-H. The Cu foam was first mechanically modified by the physical pressing method to obtain flexible dense structure, followed by subsequent surface washing to obtain clean Cu foam for further Cu@Co_3_O_4_ NWs hydrothermal assembly process. The copper foam as a substrate layer ensured the flexibility and 3D regular skeleton structure of the substrate. The smooth surface and low specific surface area of copper foam cannot meet the demand for collecting molecules to be measured, so a hydrothermal method was used to grow Co_3_O_4_ NWs on the surface of copper foam (Figure 1c and Figure S1). The dense forest-like Co_3_O_4_ NWs greatly increased the specific surface area of the substrate. There were 500–900 nm gaps between the Co_3_O_4_ NWs, which provided rich and well-spaced sites for the growth of Ag NPs and the adsorption of pesticide molecules. Loading of Ag NPs is necessary because of the higher enhancement effect than semiconductors. Next, as shown in Figure 1d, the Ag NPs were uniformly loaded on the surface of one-dimensional Co_3_O_4_ NWs and the particle size was counted to be about (23.1 ± 4.6) nm. The assembly of Ag NPs effectively utilized the 3D structure of the Cu@Co_3_O_4_ NWs to form a coral reef-like 3D hotspot space, providing the substrate with ultra-high detection performance. Figure 1e–i and Figure S2 show that EDS mapping of the substrate, which further demonstrated the success of the layer assembly and the dense distribution of Co_3_O_4_ NWs and Ag NPs.

**Figure 2:**
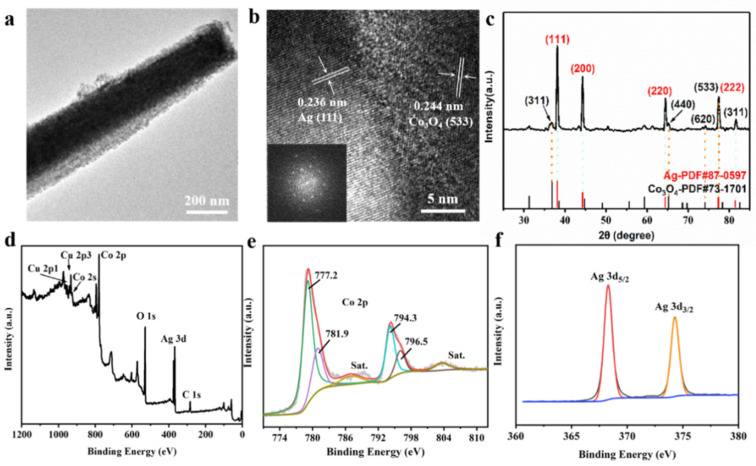
Material characterizations of the Cu@Co_3_O_4_@Ag-H substrates. (**a**) TEM image of single Co_3_O_4_ NW@Ag-H. (**b**) HRTEM image and SAED pattern of Co_3_O_4_ NW@Ag-H. (**c**) XRD pattern of Cu@Co_3_O_4_@Ag-H. (**d**) Total XPS spectrum of Cu@Co_3_O_4_@Ag-H and high-resolution XPS spectra of (**e**) Co 2p, (**f**) Ag 3d.

Figure 2a shows the TEM image of single Co_3_O_4_ NW@Ag-H, which reveals the nanowire morphology and the detailed diameter of Co_3_O_4_ was about 147 nm. Figure 2b presents the HRTEM image and SAED pattern of Co_3_O_4_ NW@Ag-H. The Ag, Co_3_O_4_ crystalline had lattice fringe spaces of 0.236 nm and 0.244 nm corresponding to the (111) and (533) lattice planes, respectively, which further manifested the validity of the effective assembly of the hierarchical substrate. X-ray diffraction (XRD) analysis of Cu foam@Co_3_O_4_@Ag-H further confirmed the existence of Ag and Co_3_O_4_ crystalline. The diffraction peaks (2θ) centered at 38.11°, 44.29°, 64.44° and 81.53° could be assigned to the (111), (200), (220) and (222) lattice planes of Ag face-center-cubic crystalline. In addition, the diffraction peaks centered at 36.84°, 65.23°, 74.12°, 77.34° and 39.39° corresponded to the (311), (440), (620), (533) and (311) lattice planes of Co_3_O_4_ orthorhombic crystal. The total XPS spectrum of Cu@Co_3_O_4_@Ag-H (Figure 2d) confirmed the above assertion. Figure 2e,f shows the high-resolution XPS profile of Co and Ag signals of substrate. Figure 2e shows that two bands at 779.5 eV and 794.6 eV, which were attributed to the Co 2p_3/2_ and Co 2p_1/2_ states of Co, respectively. Two peaks could be divided into 777.2 eV, 781.9 eV, 794.3 eV and 796.5 eV, which were attributed to the Co^3+^ and Co^2+^, confirming the formation of Co_3_O_4_ crystalline. Figure 2f displays that two bands at 374.5 eV and 368.5 eV, attributed to Ag 3d_5/2_ and Ag 3d_3/2_, respectively, and proving the formation of Ag crystalline. These values were in excellent agreement with the previous work of Zhang [39]. Figure S3a presents the two bands at 934.1 eV and 954.1 eV, with a spin energy separation of 20.0 eV, which were attributed to the Cu 2p_3/2_ and Cu 2p_1/2_ states of Cu, respectively [40,41]. Figure S3b shows the bands at 520.9 eV of O 1s, which correspond to the metal-oxygen bonds [42,43].

**Figure 3:**
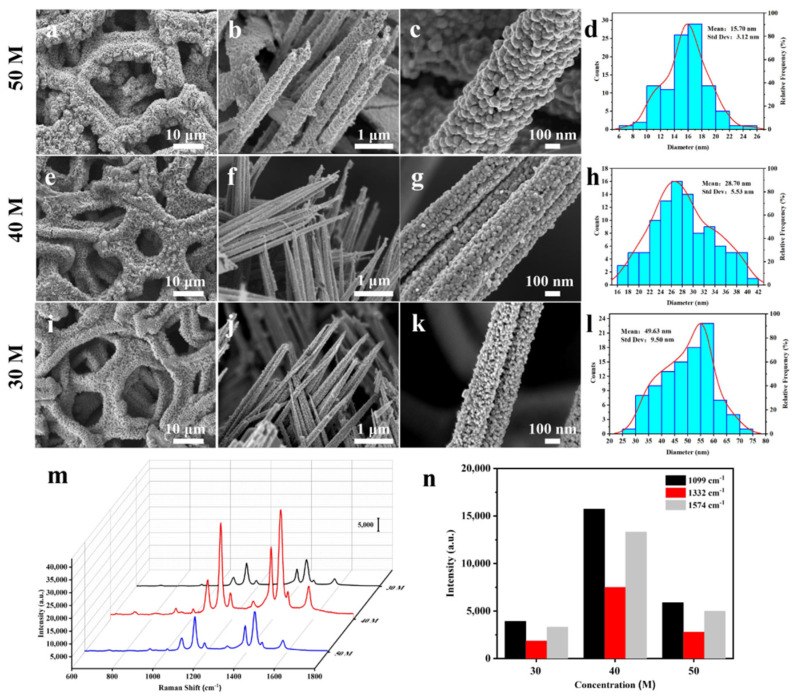
(**a**) SEM images of Cu@Co_3_O_4_@Ag-H prepared from different concentrations of silver ammonia solution (**a**–**c**) 50 M, (**e**–**g**) 40 M, (**i**–**k**) 30 M. (**d**,**h**,**l**) Corresponding Ag NPs size distribution. (**m**) Raman spectra of 10^−3^ M 4-NBT ethanol solution on three different substrates and (**n**) corresponding intensity distribution of the 1099 cm^−1^, 1332 cm^−1^ and 1574 cm^−1^ peaks.

Different strategies were made to explore how to improve SERS activity. Regulating the gap distances or sizes is the most direct means for hotspot distribution engineering for that hotspots mainly exist within 10 nm gaps between noble metal NPs [44]. The gap sizes can be effectively regulated by modulating the size and distribution of Ag NPs. In this synthesis strategy, the particle size and distribution of Ag NPs could be regulated by adjusting the concentration of silver ammonia solution while keeping the Cu@Co_3_O_4_ NWs unchanged. As shown in Figure 3, three different concentrations of silver ammonia solutions were adjusted as precursors for formation of Ag NPs on the surface of Cu@Co_3_O_4_ NWs substrates to achieve optimized morphology and hotspots distribution on Cu@Co_3_O_4_@Ag-H substrate. Figure 3i–l shows the substrate prepared by 30 M of silver ammonia solution, on which the size of Ag NPs was 15.7 nm. There are large distance gaps between nanoparticles, which was not conducive to the effective formation of hotspots induced from the EM plasmon coupling effect. Figure 3e–h presents the substrate prepared by 40 M of silver ammonia solution, on which the size of Ag NPs was 28.7 nm, and Ag NPs grew uniformly and densely on the surface of Co_3_O_4_ NW, and the narrow gaps contributed to the formation of SERS hotspots. Figure 3a–d displays the substrate prepared by 50 M of silver ammonia solution, on which the size of Ag NPs was 49.63 nm. The Ag NPs were too swollen and uneven. The SERS performance of the three substrates was then examined with 10^−3^ M of 4-NBT (Figure 3m). In Figure 3n, the characteristic peaks of 4-NBT at 1099, 1332 and 1574 cm^−1^ were selected for quantitative analysis according to Figure S4 and Table S1. Combined with the analysis of SERS properties and morphology, the optimal substrate was determined to be prepared from 40 M of silver-ammonia solution. High density gaps between Ag NPs of moderate size and tightly packed are more likely to form stronger and denser hotspots, and this has been demonstrated in a study by Puran Pandey et al. that FDTD simulations show that there are electromagnetic field (hotspots) enhancements formed by high density gaps between Ag core and Ag satellites, and between Ag satellites in PCSS nanostructures [45].

### 3.2. Preparation and Characterization of Cu@Co_3_O_4_@Ag-S

In order to compare the effect of different assembly methods of Ag on the performance of substrates, three substrates with different deposition times of Ag were prepared by physical deposition (Figure S5). Figure S5a–i illustrates that the Ag NPs obtained at three deposition times of 5 min, 10 min, and 15 min were relatively coarse, with blurred particle sizes and particle gaps, which undoubtedly led to bad SERS performance. The Raman performance tests of these three substrates also verified the above points that the substrate prepared by physical deposition had a lower Raman intensity. (Figure S5j,k). The SERS performance of substrates obtained by hydrothermal method was three times higher than that obtained by deposition method. According to the phenomenon obtained by Wang [33], the Ag NPs obtained by physical deposition methods are more densely spaced and cannot form a large number of effective hotspots, so the SERS activity of the substrate is poor. Hence, SERS substrates prepared by hydrothermal method with 40 M of silver-ammonia solution were used in this work, but physical deposition also provided a more convenient method for the preparation of SERS substrates.

### 3.3. Cu@Co_3_O_4_@Ag-H as Effective SERS Sensor

**Figure 4:**
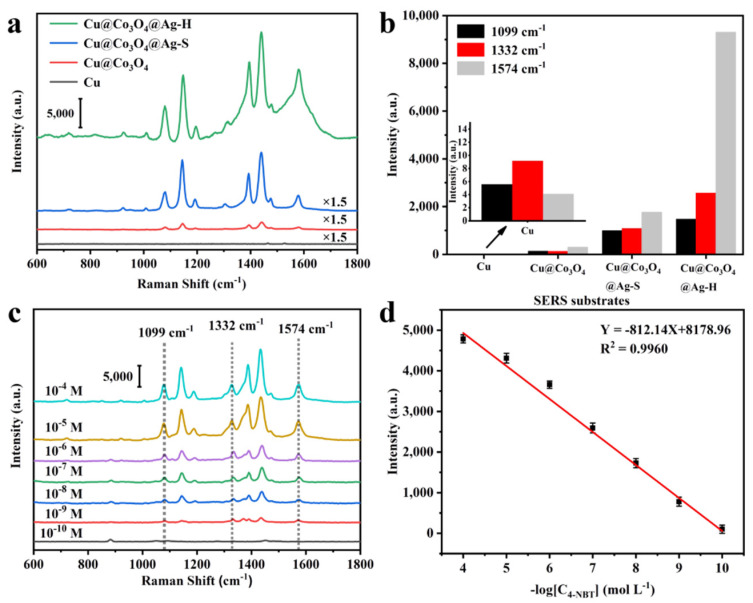
(**a**) Raman spectra of 4-NBT (10^−4^ M) on four Cu foam, Cu@Co_3_O_4_, Cu@Co_3_O_4_@Ag-S and Cu@Co_3_O_4_@Ag-H substrates for comparation and (**b**) corresponding intensity distribution of the 1099 cm^−1^, 1332 cm^−1^ and 1574 cm^−1^ peaks. (**c**) SERS spectra of 4-NBT on the Cu@Co_3_O_4_@Ag-H substrate at different concentrations from 10^−4^ M to 10^−10^ M. (**d**) Relationship between the intensity of the SERS peak at 1332 cm^−1^ and the 4-NBT concentration; the error bar is based on four parallel SERS spectra.

**Figure 5:**
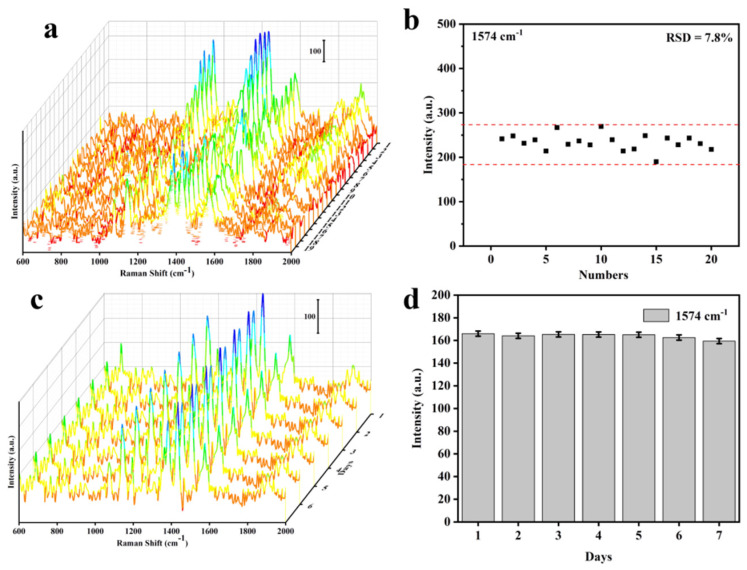
Raman spectra of 10^−5^ M 4-NBT on the Cu@Co_3_O_4_@Ag-H substrate. (**a**,**b**) Raman spectra waterfall plots with 20 points randomly selected from the mapping spectra and corresponding intensity distribution of the 1574 cm^−1^ peaks of 4-NBT collected for RSD analysis. (**c**,**d**) Raman spectra waterfall plots of the 4-NBT on the Cu@Co_3_O_4_@Ag-H substrate at different collection times and corresponding intensity variation of the 1574 cm^−1^ peaks of 4-NBT collected for intensity reduction analysis.

Figure 4a displays the SERS performance of Cu foam, Cu@Co_3_O_4_ NWs, Cu@Co_3_O_4_@Ag-S and Cu@Co_3_O_4_@Ag-H substrates. By comparing the characteristic peaks of 1099, 1332 and 1574 cm^−1^, the superiority of the Cu@Co_3_O_4_@Ag-H substrate was obvious (Figure 4b). Comparing Cu and Cu@Co_3_O_4_ NWs, it could not be ignored that Cu@Co_3_O_4_ NWs substrate could also detect the characteristic peaks of 4-NBT, which demonstrated that Co_3_O_4_ NWs had efficient enrichment capacity and provided a contribution to the SERS enhancement of the Cu@Co_3_O_4_@Ag substrate. The SERS activity of both Cu@Co_3_O_4_@Ag-S and Cu@Co_3_O_4_@Ag-H was superior to that of Cu foam@Co_3_O_4_ NWs, demonstrating that the EM effect of Ag NPs provide a much higher SERS enhancement than that of Cu foam from CM effect. Then, the SERS performance of the substrates was examined using different concentrations of 4-NBT from 10^−4^ M to 10^−10^ M (Figure 4c). The substrate was able to detect the 4-NBT even at concentrations as low as 10^−10^ M, and the detection signal maintained great linear correlation (R^2^ = 0.9960) in this concentration range from 10^−4^ M to 10^−10^ M (Figure 4d). The SERS enhancement factor (EF) was estimated as ~2.24 × 10^−8^ (details see Figure S6 in SI), which demonstrated the ultra-high sensitivity of the substrate for the detection of trace molecules. To examine the uniformity of the SERS sensor, we randomly took 20 sites for evaluation (Figure 5a). After statistical analysis, RSD of the SERS intensity variation of 1574 cm^−1^ was only ~7.8% (Figure 5b). Then, in order to detect the stability of the substrate, we collected the SERS signal of 4-NBT at the same point repeatedly over 7 days (Figure 5c). By counting the intensity of the 1574 cm^−1^ of 4-NBT, it was found that the signal of 4-NBT lost only 5.7% on the Cu@Co_3_O_4_@Ag-H substrate after 7 days (Figure 5d). All of the above findings demonstrated that the Cu@Co_3_O_4_@Ag-H substrates possess excellent SERS performance. These results proved that the prepared Cu@Co_3_O_4_@Ag-H substrate is available for label-free detection of ultrasensitive, quantitative, stable and homogeneous SERS sensing and has the potential for practical in-situ detection monitoring of real-world complex environments.

### 3.4. Trace Droplet Rapid Cu@Co_3_O_4_@Ag-F Sensing Platform

**Figure 6:**
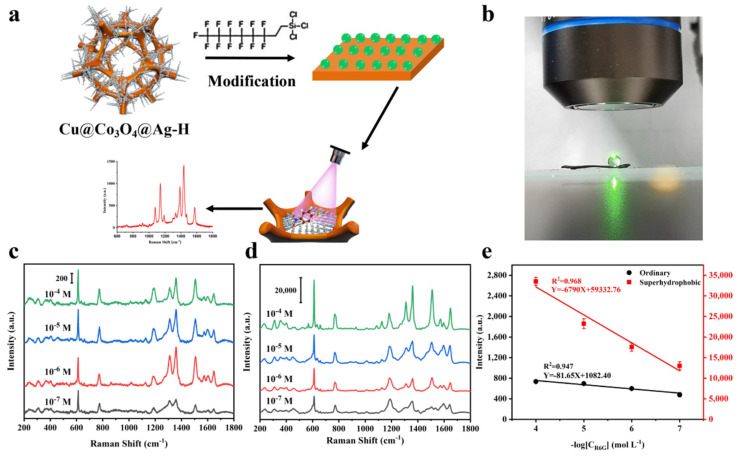
(**a**) Schematic illustration of the fabrication process toward Cu@Co_3_O_4_@Ag-F SERS sensor. (**b**) Image of superhydrophobic property of the Cu@Co_3_O_4_@Ag-F substrate. (**c**,**d**) Raman spectra of R6G from 10^−4^ M to 10^−7^ M on Cu@Co_3_O_4_@Ag-H substrate (**c**) and Cu@Co_3_O_4_@Ag-F substrate (**c**). (**e**) Relationship between the intensity of the SERS peak at 612 cm^−1^ and the R6G concentration; the error bar is based on four parallel SERS spectra.

For analyte as low as pico-m or femto-m, the target molecules to be detected are usually sparsely distributed in water, which leads to the difficulty of low activity in Raman detection experiment. In order to further expand the application flexibility of the Cu@Co_3_O_4_@Ag platform, the trace detection ability is further developed according to hydrophobic modification of the substrate. As depicted in Figure 6a, superhydrophobic Cu@Co_3_O_4_@Ag-F SERS substrate was prepared from Cu@Co_3_O_4_@Ag-H with surface treatment with FAS-17 surfactant. With FAS-17 having low surface energy terminal -CF_3_ group and -CF_2_ group, FAS-17 are able to effectively reduce the substrate surface energy and successfully induce hydrophobic modification of the substrate [46]. Figure S7 shows that the contact angle (CA) of the droplet on the substrate changed dramatically after the hydrophobic modification, CA changed from 60° to 136°, and the substrate had hydrophobic properties. Figure 6b shows an optical photograph of the droplet in the actual Raman detection, which also exhibits the superhydrophobic properties of the substrate. To verify the better detection ability of the Cu@Co_3_O_4_@Ag-F substrate, ordinary substrate without surface hydrophobic modification was compared as the control group. Ten microliters of different concentration (10^−4^–10^−7^ M) of R6G aqueous solution were dipped onto the two substrate surfaces for Raman signal acquisition (Figure 6c,d), The Raman spectra and detailed Raman band assignments of R6G were shown in Figure S8 and Table S2, respectively. By counting the characteristic peak at 612 cm^−1^ (δ(C-C) _ring_) of R6G, it was found that the substrate could achieve a nearly 5-fold improvement in detection performance by hydrophobic treatment than that of hydrophilic ordinary substrate. Besides, the detection signal maintained greater linear correlation (R^2^ = 0.968) from 10^−4^ M to 10^−7^ M (Figure 6e) on Cu@Co_3_O_4_@Ag-F substrate. Consequently, the trace detection ability is achieved on Cu@Co_3_O_4_@Ag-F substrate for real rapid analyst application.

### 3.5. Real Application of Fruits and Vegetables Thiram SERS Sensing

**Figure 7:**
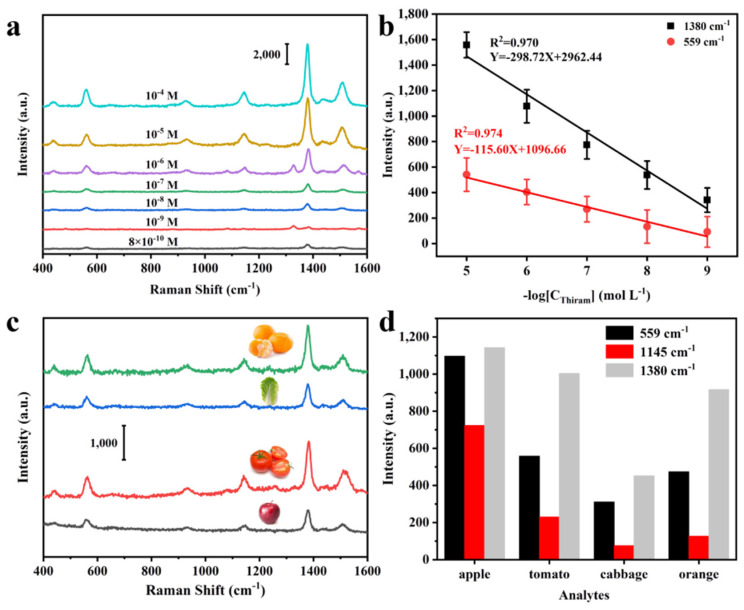
Raman spectra of thiram on the Cu@Co_3_O_4_@Ag-H substrate. (**a**) SERS spectra of thiram at different concentrations from 100 ppm to 800 ppt. (**b**) Relationship between the intensity of the SERS peak at 559 cm^−1^, 1380 cm^−1^ and the thiram concentration; the error bar is based on four parallel SERS spectra. (**c**) Raman spectra and (**d**) corresponding intensity of 559 cm^−1^,1145 cm^−1^, 1380 cm^−1^ of thiram (100 ppb) collected from four different fruits and vegetables.

The original purpose of pesticides is to protect the normal growth of plants and eliminate pests, but the intake of fruits and vegetables with high concentrations of pesticide residues can also be seriously harmful to humans. Current detection methods for pesticides mostly have limited detection concentration, long detection time, high detection costs, complex detection pre-treatment and other problems. The emergence of SERS detection means a suitable solution to several of them, but the high cost of detection and complex pre-treatment have been a major problem of pesticide residue detection. Herein, these problems were effectively solved by utilizing the Cu@Co_3_O_4_@Ag-H SERS substrates. The adsorption ability of pesticide molecules on porous Cu@Co_3_O_4_@Ag-H SERS sensor was firstly assessed. Figure S9 illustrates the adsorption curves of substrates for pesticide molecules at different concentrations, according to the data fit analysis, which is well consistent with the Langmuir adsorption model. Due to the enormous specific surface area, the maximum adsorption capacity of Cu@Co_3_O_4_@Ag-H for thiram reached 483.23 μg·cm^−2^. At the same time, it takes only 2 s to achieve effective enrichment of pesticide molecules in solution, so it is of great significance in actual pesticide residue rapid detection. Figure 7a demonstrates the Raman spectra of different concentrations of thiram solutions. According to the standard Raman spectra and the detailed Raman band assignments of thiram given in Figure S10 and Table S3, even down to 800 ppt, the Raman peak at 1380 cm^−1^ (CH_3_ symmetric rocking, C–N stretching) was still detectable, which was sufficient to prove the excellent detection ability of the Cu@Co_3_O_4_@Ag-F substrate. Figure 7b illustrates that the Raman intensity of thiram maintains a good linearity (R^2^ = 0.970, R^2^ = 0.974) in the concentration range from 10^−5^ M to 10^−9^ M, enabling the qualitative and quantitative detection of thiram at ultra-low concentrations.

To simulate the detection of pesticide residues on the surface of fruits and vegetables in real situations, as well as to verify the “wipe” detection capability of the substrate, a new series of experiments were conducted. The detection results are shown in Figure 7c. with clear characteristic peaks at 559, 1145 and 1380 cm^−1^ of thiram being detected. Although the different roughness of the selected fruits and vegetables surfaces led to different detection intensities, the Cu@Co_3_O_4_@Ag-H substrate could still clearly detect the characteristic peaks of 100 ppb of thiram. Thus, the Cu@Co_3_O_4_@Ag-H SERS sensor is available for effective rapid detection of thiram at 0.24 ng cm^−^^2^ (the ng·cm^−^^2^ values are based on the geometrical area and not surface area), which is lower than the reported works as summarized in Table 1. The test within 2s confirms the rapid effective availability of Cu@Co_3_O_4_@Ag-H SERS sensor, which offers new potential for bioassay, disease POCT diagnosis, national security, energy storage and related applications.

## 4. Conclusions

In conclusion, an innovative 3D plasmonic hierarchical porous Cu@Co_3_O_4_@Ag-F array was firstly developed with controllable Ag NPs sizes and deposition density for effective plasmon coupling effect by a two-step hydrothermal reduction synthesis strategy. The strategy offers a new avenue for localized functional materials distribution and construction, which exhibits better morphology regulation ability and SERS activity than physical spurring obtained Cu@Co_3_O_4_@Ag-S. The Cu@Co_3_O_4_@Ag-H not only provides EM hotspots but also exhibit the trace detection capability for droplet rapid sensing by hydrophobic modification. System evaluation results proved that the prepared Cu@Co_3_O_4_@Ag-H substrate is available for label-free detection of ultrasensitive, quantitative, stable, trace droplet rapid capability and homogeneous SERS sensing ability, which exhibits potential for practical in-situ detection monitoring of real-world complex environments. Meanwhile, highly sensitive qualitative and quantitative thiram detection in real application on fruits and vegetables is achieved, which demonstrates the universality of the high throughput SERS sensor. This finding will offer new potential for bioassay, disease POCT diagnosis, national security, and related applications.

## Figures and Tables

**Table 1:** SERS performance of thiram detection on different SERS substrates.

Object	SERS Substrates	Target	LOD	References
apples, pears, grapes	Fe_3_O_4_@graphene oxide@Ag	thiram	0.48 ng·cm^−2^	[47]
cucumber	3D PDMS Nanotentacle array with Ag NPs	thiram	1.6 ng·cm^−2^	[33]
lemon	Silicon nanowire paper modified with Au NPs	thiram	72 ng·cm^−2^	[8]
oranges, green vegetables	“Paste and peel” Au NPs	thiram	2.6 ng·cm^−2^	[26]
grape juice	Ag dendritic nanostructures	thiram	0.03 ppm	[48]
apple juice	PMMA/Ag NPs/graphene	thiram	0.24 ppm	[49]
orange, cabbage, tomato and apple	3D hierarchical porous Cu@Co_3_O_4_@Ag-H	thiram	0.24 ng·cm^−2^ (0.1 ppm)	This work

## Data Availability

The data presented in this study are available on request from the 251 corresponding author.

## Supplementary Materials

The following are available online at https://www.mdpi.com/article/10.3390/nano11123460/s1. Figure S1. SEM images of Cu@Co_3_O_4_ NWs under different magnifications. Figure S2. EDS spectrum of hierarchical Cu@Co_3_O_4_@Ag-H substrates. Figure S3. High-resolution XPS spectra of (a) Cu 2p and (b) O 1s of Cu@Co_3_O_4_@Ag-H substrates. Figure S4. Raman spectrum of 4-NBT (10^−5^ M). Table S1. The detailed Raman band assignment of 4-NBT. Figure S5. (a) SEM images of Cu@Co_3_O_4_ @Ag-S prepared at different deposition times (a–c) 5 min, (d–f) 10 min, (g–i) 15 min, (j) Raman spectra of 10^−3^ M 4-NBT ethanol solution on different substrates with deposition times of 5 min, 10 min and 15 min, respectively, and (k) Corresponding intensity distribution of the 1099 cm^−1^, 1332 cm^−1^ and 1574 cm^−1^ peaks. Figure S6. Raman spectra of 4-NBT for EF measurement. Figure S7. Water contact angle on Cu@Co_3_O_4_@Ag-F substrate (a) Before superhydrophobic treatment; (b) After superhydrophobic treatment. Figure S8. Raman spectrum of R6G (10^−4^ M). Table S2. The detailed Raman band assignment of R6G. Figure S9. Adsorption curves of thiram at different concentrations on the Superhydrophobic Cu@Co_3_O_4_@Ag-H substrates. Figure S10. Raman spectrum of thiram (100 ppm). Table S3. The detailed Raman band assignment of thiram.
