# Supplementary material for: A Novel 3D Hierarchical Plasmonic Functional Cu@Co3O4@Ag Array as Intelligent SERS Sensing Platform with Trace Droplet Rapid Detection Ability for Pesticide Residue Detection on Fruits and Vegetables
Source: Nanomaterials (Basel). 2021 Dec 20;11(12):3460. doi: 10.3390/nano11123460 (PMC8705477; doi:10.3390/nano11123460)
Supplement: Supplementary file 1 [file nanomaterials-11-03460-s001.zip › nanomaterials-1504092-supplementary.pdf]

nanomaterials

*Supporting Information*

# A Novel 3D Hierarchical Plasmonic Functional Cu@Co3O4@Ag Array as Intelligent SERS Sensing Platform with Trace Droplet Rapid Detection Ability for Pesticide Residue Detection on Fruits and Vegetables

**Guanliang Sun [a,†], Ning Li [a,b,†], Dan Wang [a], Guanchen Xu [a], Xingshuang Zhang [a], Hongyu Gong [a], Dongwei Li [a], Yong Li [a], Huaipeng Pang [a], Meng Gao [a] and Xiu Liang [a,*]**

a Key Laboratory for High Strength Lightweight Metallic Materials of Shandong Province (HM),Advanced Materials Institute, Qilu University of Technology (Shandong Academy of Sciences), Jinan 250014, P. R. China

b Center of Excellence for Environmental Safety and Biological Effects, Beijing Key Laboratory for Green Catalysis and Separation, Department of Chemistry and Biology, Beijing University of Technology, Beijing, 100124, P. R. China

* Corresponding author at: Advanced Materials Institute, Qilu University of Technology (Shandong

Academy of Sciences), Jinan, Shandong 250014, P. R. China;

E-mail address: *xliang@sdas.org (Xiu Liang)*

† These authors contributed equally to this work.

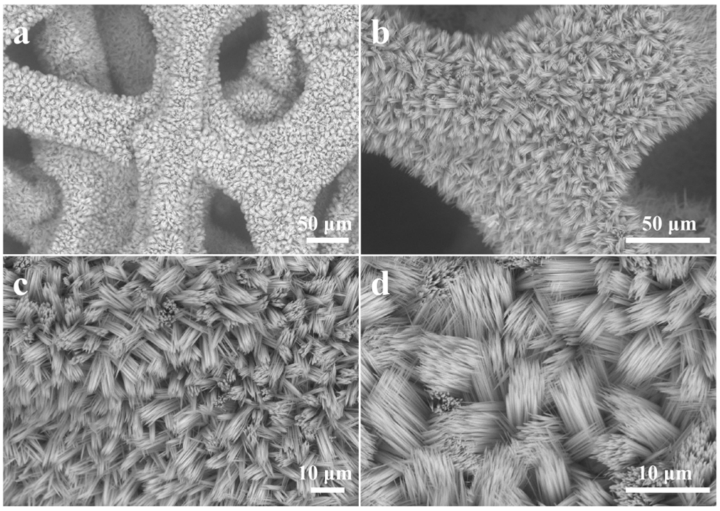


**Fig S1.** SEM images of Cu@$Co_3O_4$ NWs under different magnifications.

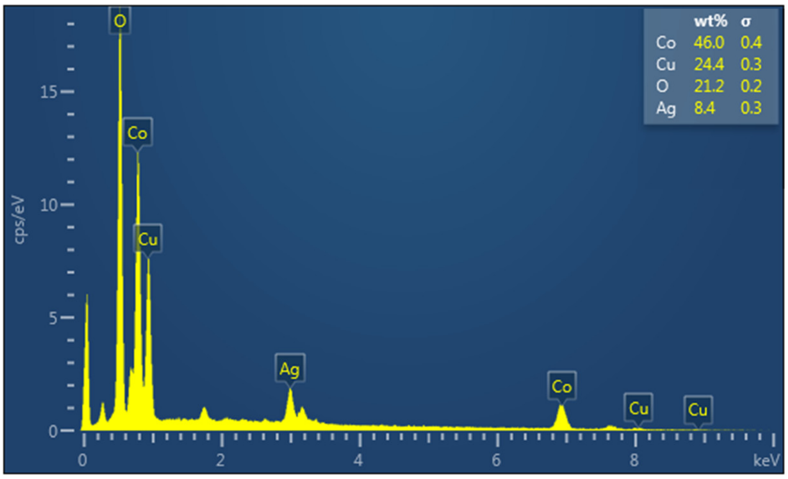


**Fig S2.** EDS spectrum of hierarchical Cu@$Co_3O_4$@Ag-H substrates.

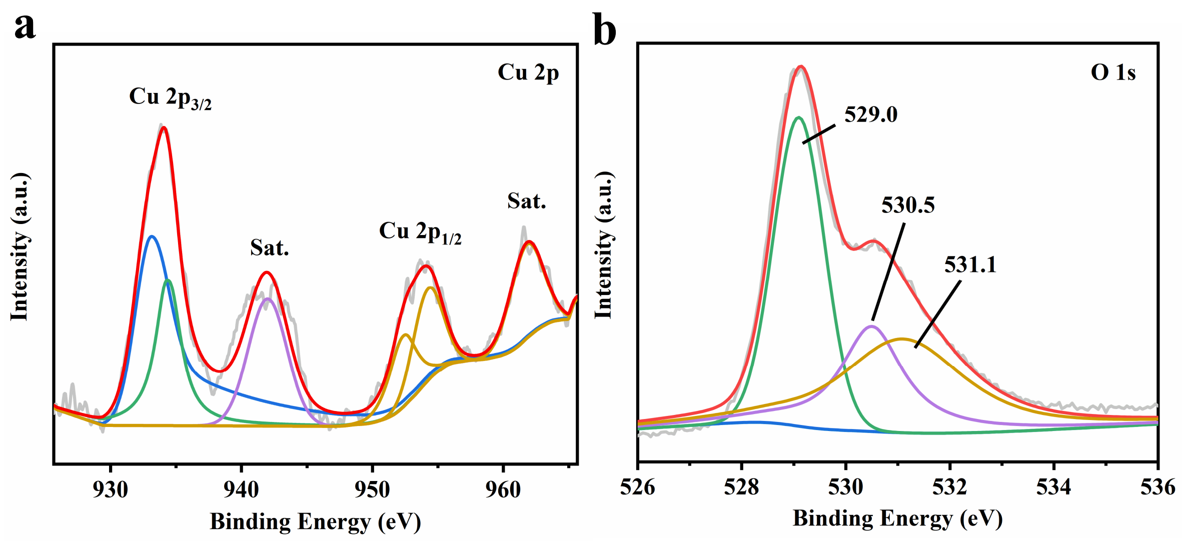


**Fig S3.** High-resolution XPS spectra of (a) Cu 2p and (b) O 1s of Cu@$Co_3O_4$@Ag-H substrates.

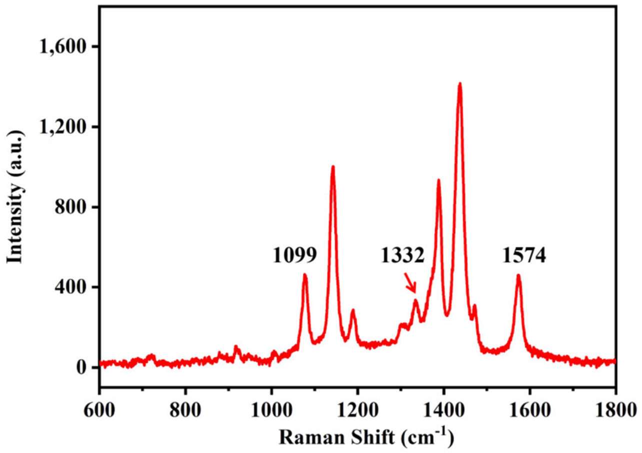


**Fig S4.** Raman spectrum of 4-NBT ($10^{-5}$ M).

**Table S1. The detailed Raman band assignment of 4-NBT.**

| Observed band ($cm^{-1}$) | Vibrational mode |
|---|---|
| 1099 | $CH_3NC$ deformation, C=S stretching |
| 1332 | S-S stretching |
| 1574 | $CH_3N$ stretching, C=S stretching |

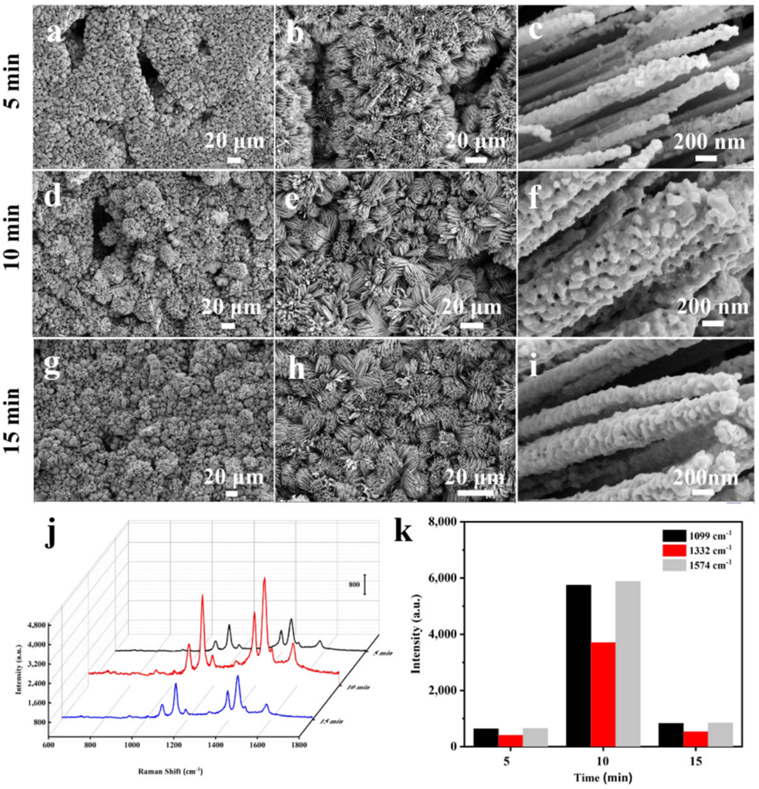


**Fig S5.** (a) SEM images of Cu@$Co_3O_4$ @Ag-S prepared at different deposition times (a-c) 5 min, (d-f) 10 min, (g-i) 15 min. (j) Raman spectra of $10^{-3}$ M 4-NBT ethanol solution on different substrates with deposition times of 5 min, 10 min and 15 min, respectively. and (k) Corresponding intensity distribution of the 1099 $cm^{-1}$, 1332 $cm^{-1}$and 1574 $cm^{-1}$ peaks.

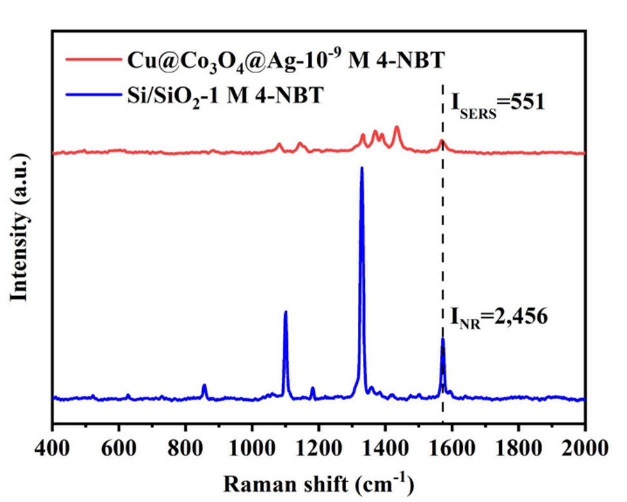


**Fig S6. Raman spectra of 4-NBT for EF measurement:** red line presents SERS spectrum of $10^{-9}$ M 4-NBT absorbed on the SERS substrate (multiplied by 5); blue line presents Raman spectrum of 1 M 4-NBT absorbed on the blank non-SERS platform ($Si/SiO_2$ wafer).

The enhancement factor (EF) of the as-prepared SERS substrate was estimated according to the widely accepted formula:

$$EF = \frac{I_{SERS}/N_{SERS}}{I_{NR}/N_{NR}}$$

The $I_{SERS}$ and $I_{NR}$ are the intensities of the selected band in the SERS and normal Raman spectra, respectively. $N_{SERS}$ and $N_{NR}$ are the estimated molecule number under laser excitation for SERS, and the molecule number for the reference sample (solid), respectively.

We take the intensity at the characteristic peak 1574 $cm^{-1}$ of 4-NBT as the value of $I_{SERS}$ and $I_{NR}$. We can see $I_{SERS}$ =551 (multiplied by 5), $I_{NR}$ =2513 (multiplied by 5) from the **Fig S6**, and simplify $N_{SERS}$ and $N_{NR}$ to the concentration of 4-NBT solution dripping on SERS substrate and $Si/SiO_2$ wafer substrate. EF=$2.24\times10^8$ can be obtained by calculation.

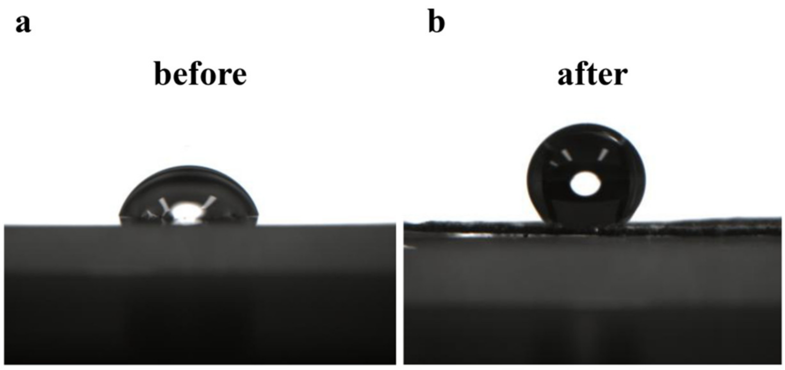


**Fig S7.** Water contact angle on Cu@$Co_3O_4$@Ag-F substrate (a) Before superhydrophobic treatment; (b) After superhydrophobic treatment.

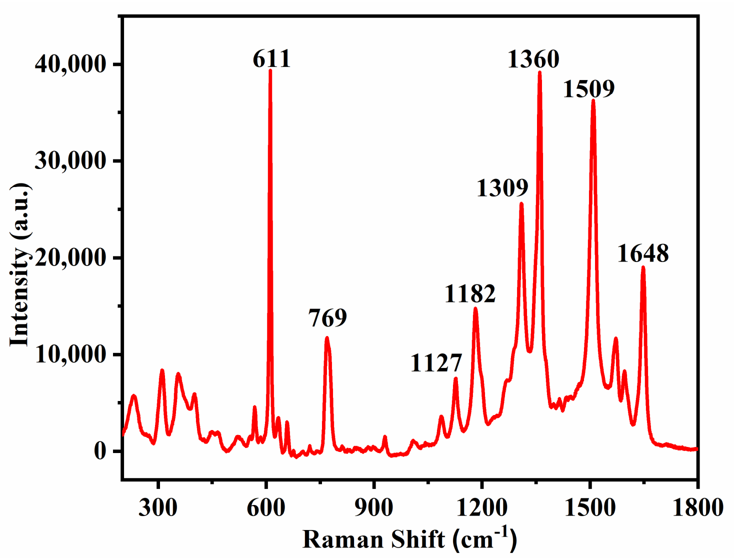


**Fig S8.** Raman spectrum of R6G ($10^{-4}$ M).

**Table S2. The detailed Raman band assignment of R6G.**

| Observed band ($cm^{-1}$) | Vibrational mode |
|---|---|
| 612 | C-C in plane deformation modes $\delta$(C-C) $_{ring}$ |
| 769 | C-H out of plane deformation modes $\delta$ (C-H) $_{ring}$ |
| 1182 | C-H and N-H bending modes $\beta$(C-H) $_{xanthene}$ and $\beta$(C-H) $_{xanthene}$ |

| | |
|---|---|
| **1309** | **C=C stretching modes ν(C=C)** $_{xanthene}$ |
| **1509** | **C-C stretching modes ν(C-C)** $_{xanthene}$ |
| **1648** | **C=O stretching modes ν(C=O)** |

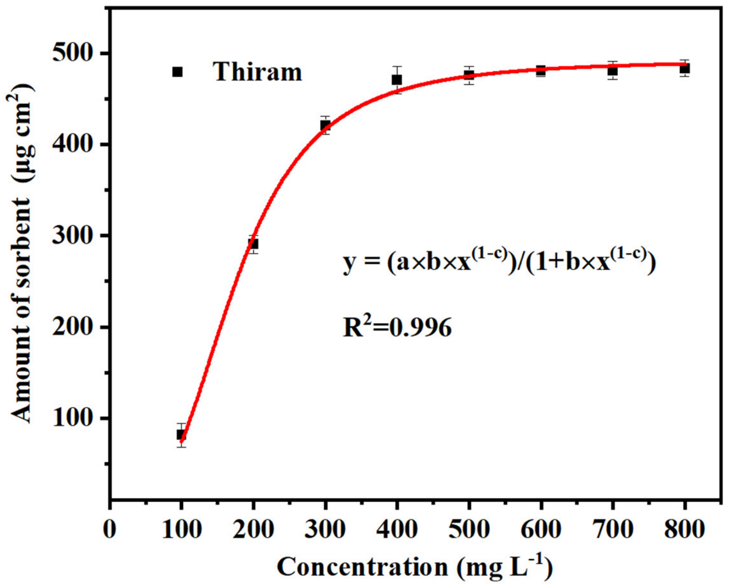


**Fig S9.** Adsorption curves of thiram at different concentrations on the superhydrophobic $Cu@Co_3O_4@Ag$-H substrates.

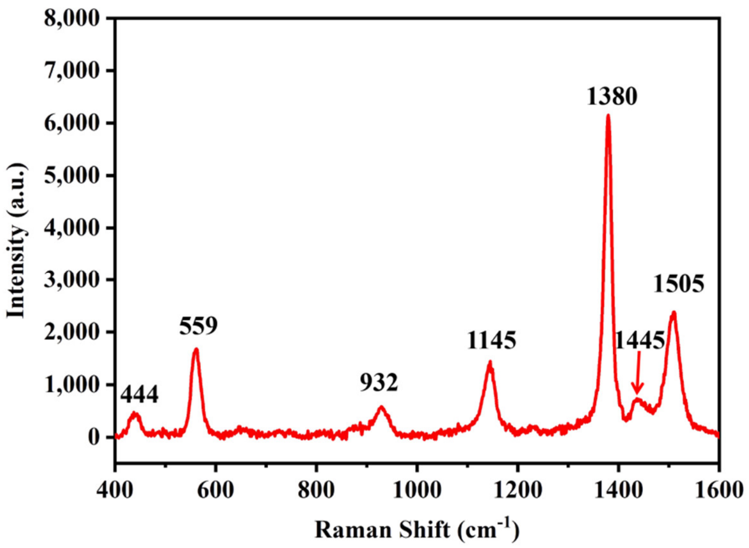


**Fig S10.** Raman spectrum of thiram (100 ppm).

**Table S3. The detailed Raman band assignment of thiram.**

| **Observed band ($cm^{-1}$)** | **Vibrational mode** |
|---|---|
| **444** | **$CH_3NC$ deformation, C=S stretching** |
| **559** | **S-S stretching** |
| **932** | **$CH_3N$ stretching, C=S stretching** |
| **1145** | **$CH_3$ rocking, C-N stretching** |
| **1380** | **$CH_3$ symmetric rocking, C-N stretching** |
| **1445** | **$CH_3$ antisymmetric deformation** |
| **1505** | **$CH_3$ rocking, C-N stretching** |
